# Toxicokinetics of Chromium in *Enchytraeus crypticus* (Oligochaeta)

**DOI:** 10.3390/toxics10020082

**Published:** 2022-02-09

**Authors:** Fátima C. F. Santos, Rudo A. Verweij, Cornelis A. M. van Gestel, Mónica J. B. Amorim

**Affiliations:** 1 Department of Biology & CESAM, University of Aveiro, 3810-193 Aveiro, Portugal; fatimasantos@ua.pt; 2 Department of Ecological Science, Faculty of Science, Vrije Universiteit Amsterdam, De Boelelaan 1085, 1081 HV Amsterdam, The Netherlands; rudo.verweij@vu.nl (R.A.V.); kees.van.gestel@vu.nl (C.A.M.v.G.)

**Keywords:** trivalent chromium, enchytraeids, bioaccumulation, uptake, elimination

## Abstract

Chromium is naturally occurring, but emission from anthropogenic sources can lead to increased soil concentrations. Information on its toxicokinetics is essential in order to understand the time needed to reach toxicity and the mechanisms of uptake/elimination. In this study the toxicokinetics of Cr(III) was evaluated using the soil standard species *Enchytraeus crypticus*. The animals were exposed to 180 mg Cr/kg dry soil, a sublethal concentration, in LUFA 2.2 natural soil. OECD guideline 317 was followed, with a 14-day uptake phase in spiked soil followed by a 14-day elimination in clean soil. Exposure to Cr led to fast uptake and elimination, with *K_u_* = 0.012 kg_soil_/kg_organism_/day and *K_e_* = 0.57 day^−1^. The bioaccumulation factor was 0.022, and DT_50_ for elimination was 1.2 days. The concentration of Cr reached an internal equilibrium in the animals after 10 days. Transfer to clean soil allowed body Cr concentrations to return to background levels after approximately 7 days. *E. crypticus* seemed able to efficiently regulate internal Cr concentrations by actively eliminating Cr (an essential element). Although *K_u_* and *K_e_* deviated from the values reported in other studies for other soil invertebrates, the bioaccumulation factors were similar. These findings show the importance of toxicokinetic studies in evaluating toxicity based on internal metal concentrations that can more accurately represent the bioavailable concentration.

## 1. Introduction

The naturally occurring element chromium (Cr) is present in soils, plants, and animals, as well as in other environmental compartments [1,2]. The main contributions to Cr contamination in the environment are anthropogenic sources like emissions from metal smelters, industrial wastes and spills, and landfills [2,3,4,5,6]. The anthropogenic use of Cr includes organic fertilizers, leather tanning, application of Cr-containing compounds such as pigments, chromates, and corrosion inhibitors, among others [1,7]. The use of Cr may result in high levels of Cr in soil, potentially affecting biota [8]. 

Chromium exists in different oxidation states [9,10]. Chemical speciation affects the biogeochemical cycle of Cr, with Cr(III) being less soluble, toxic, and mobile than Cr(VI) [4,11]. High levels of Cr(VI) may seriously affect microbial activity, leading to soil deterioration, which in turn leads to a reduction in plant productivity [12]. Cr(VI) contamination is also associated with specific forms of cancer, allergic reactions, neurological and cardiovascular diseases, and organ failure [2,4,12]. Cr(III) is an essential component of human and animal nutrition and health when in trace amounts [4,11,13]. In soil, the dominant Cr form is Cr(III) [14]. 

Survival and reproduction are the most studied endpoints when assessing the effects of Cr on soil organisms such as enchytraeids, earthworms, and springtails [8,15,16,17,18,19,20,21]. Enchytraeids present a ubiquitous distribution and large abundance in most soils and different ecosystems, being one of the most relevant soil organisms [22]. They play crucial functions in soil ecosystems, including soil bioturbation, helping the decomposition of organic matter and improving the overall quality of the soil, especially at high population densities [15,23].

Toxicokinetics enables the description of the time course of metal accumulation from external into internal concentration considering the factor time and including ADME (absorption, distribution, metabolism, and excretion) [24,25]. A toxicokinetic approach has the advantage of quantification of the internal concentration at different points in time, regardless of the exposure conditions (constant or pulse) or other environmental factors, and may help explain the toxic effects observed [26]. In our study the abiotic factors and the exposure were constant over time, so we do not expect these factors to influence the toxicokinetic modelling. 

Toxicokinetic studies with Cr and soil invertebrates exist but are scarce. Kilpi-Koski et al. [27] exposed the earthworm *Eisenia andrei* for 42 days to high, medium, and low chromated copper arsenate (CCA)-contaminated soils where the measured concentrations varied between 12.5 and 1592 mg Cr/kg. The uptake was very fast, reaching a steady state after just one day, with bioaccumulation factors (BAFs) of 0.029, 0.036. Van Gestel et al. [28] performed a study exposing *E. andrei* in artificial soil to Cr for 3 weeks, and reported internal concentrations between 0.8 and 18 mg Cr/kg dry weight. No studies were found on the toxicokinetics of Cr in the enchytraeid worm *Enchytraeus crypticus*, but other literature studies have evaluated the toxicokinetics of different metals using different soil model organisms: *E. crypticus* exposed to Ni [29], to Cu and Cd [30], to Pb [31], and to Ag [32]; and *Eisenia fetida* exposed to Cu and Cd [33]. 

The aim of this study was to evaluate the toxicokinetics of the essential element Cr in *E. crypticus*, a standard soil model species. LUFA 2.2 soil was used, and we exposed the organisms to the concentration causing 10% reduction in the number of juveniles produced (180 mg Cr/kg) [34]. Uptake and elimination were followed over 28 days of exposure. We hypothesized that *E. crypticus* can take up and eliminate chromium by regulating its internal concentration. 

## 2. Materials and Methods

### 2.1. Test Organism

*Enchytraeus crypticus* (Enchytraeidae, Oligochaeta) [35] was used. Cultures were kept in agar plates consisting of Bacti-agar medium (Oxoid, Agar No. 1) and a mixture of 4 different salt solutions with final concentrations of 0.75 mM NaHCO_3_, 0.08 mM KCl, 2 mM CaCl_2_·2H_2_O, and 1 mM MgSO_4_. Cultures were fed ad libitum on ground oats twice per week and kept at 19 ± 1 °C and a 16/8 h light/dark photoperiod. Test animals were selected among adults with well-developed clitellum. 

### 2.2. Test Soil 

LUFA 2.2 soil (Speyer, Germany) was used. Soil characteristics included 5.6 pH (0.01 M CaCl_2_), 1.71% organic carbon, 9.2 cmol_c_/kg CEC (cation exchange capacity), 44.8% WHC (water holding capacity), and grain size distribution of 8.0% clay, 13.7% silt, and 78.3% sand. Before use, the soil was dried at 80 °C for 48 h.

### 2.3. Test Substance and Spiking Procedures

Soil was spiked by adding CrCl_3_·6H_2_O (Fisher Chemical, CAS: 10060-12-5; Extra Pure) dissolved in water to achieve the nominal concentration of 180 mg Cr/kg soil DW (dry weight). This concentration was based on the EC_10_ of 180 mg Cr/kg for effects on enchytraeid reproduction obtained in a standard reproduction toxicity test [34]. Prior to test start, soil was moistened at 50% of the WHC and left to equilibrate for 14 days at 19 ± 1 °C. 

### 2.4. Experimental Procedure 

OECD guideline 317 for bioaccumulation in terrestrial oligochaetes was followed [36]. At test start, in each replicate test vessel containing 20 g moist soil and food (15 mg of autoclaved oats), 10 adult enchytraeids were introduced. After the uptake phase (14 days in the spiked soil), surviving animals were transferred to clean soil to evaluate the elimination phase (14 days in clean soil). Sampling of 5 replicates was done at 7 time points for the uptake phase (days 0, 1, 2, 4, 7, 10, 14) and 6 time points for the elimination phase (days 15, 16, 18, 21, 24, 28) to analyze internal Cr concentration. At days 0, 14, and 28, controls with non-spiked soil were also sampled. Food (15 mg of oats) and water (based on weight loss) were renewed weekly, and the tests ran at 20 ± 1 °C and a 16/8 h light/dark photoperiod.

Upon sampling, the content of a replicate test vessel was transferred to a Petri dish (no water added) and the soil was searched for the 10 enchytraeids with the help of a brush and soft tweezers (so as not to harm the animals). The collected animals from each replicate were transferred to 24-well plates with ISO water [37] for a period of 8 h to allow them to depurate their gut of soil particles. Five animals from each replicate exposed and control test vessel (at test start containing 10 individuals) were introduced into cryotubes individually for later measurement of their internal Cr concentration. For day 0 measurements, animals were collected directly from the laboratory cultures. 

Samples were frozen at −20 °C until analysis. After collecting the enchytraeids, the soil was dried for 48 h at 40 °C and stored at −20 °C for analysis. At day 0, soil for analysis was taken from the spiked and control batches. 

### 2.5. Chemical Analysis

Animal samples were freeze-dried for 24 h, weighed on a microbalance (individually), and with the addition of 300 µL of a 1:7 (*v*/*v*) mixture of HClO_4_ (70%; J.T.Baker (Avantor) Ultrex Ultra-Pure, Radnor, PA, USA) and HNO_3_ (65%; Fischer Scientific OPTIMA Grade, Loughborough, UK) were digested in a block heater (Techne Dri-Block Heater, Staffordshire, UK) using increasing temperatures ranging between 85 and 180 °C. After evaporation of the acid, the residues were taken up in 0.1 M HNO_3_, and the Cr concentrations in the digests were measured by graphite furnace atomic absorption spectrometry (AAS; PinAAcle 900Z, Perkin Elmer, Singapore). No certified reference material was available to check for the quality of the analysis. Limit of detection (LOD) for Cr was 0.0009 µg/L. 

For soil total concentration measurements, 130 mg dry soil samples from 3 out of the 5 replicates available per sampling day were heated with a 1:4 (*v*/*v*) destruction mixture (2 mL) of HCl (37%, Merck Emsure, Darmstadt, Germany) and HNO_3_ (65%, Merck Emparta, Darmstadt, Germany) in a tightly closed Teflon container at 140 °C for 7 h. After cooling, 8 mL of deionized water was added, and the metal concentrations were measured by flame atomic absorption spectrometry (AAS, AAnalyst 100; Perkin Elmer, Bodenseewerk, Germany). Total soil concentrations were also measured for the elimination phase (at day 15 and day 28). Certified reference material WEPAL was used as quality control; mean (±SD; *n* = 2) Cr reference material concentrations measured were 144 ± 1.77% of the certified value. 

To determine CaCl_2_ extractable Cr concentration, 25 mL of 0.01 M CaCl_2_ solution was added to 5 g of soil and shaken for 2 h at 200 rpm. After allowing sedimentation overnight, pH was measured, and the supernatant was filtered over 0.45 µm nylon syringe filters. The metal concentration in the 0.01 M CaCl_2_ extracts was measured by flame AAS (AAnalyst 100; Perkin Elmer; Germany). 

### 2.6. Data Analysis 

A one-compartment model was used to describe Cr uptake and elimination in the animals, assuming constant exposure concentrations [38] and simultaneously fitting the following equations 1 and 2 to data from the uptake and elimination phase. 

Uptake phase:

(1) $$C_{t}=C_{0}\;+\frac{K_{u}}{\left(K_{e}\;+\;K_{growth}\right)}\;*\;C_{exp}\;*\left(1\;-e^{-\left(K_{e}\;+\;K_{growth}\right)\;*\;t}\right)$$

Elimination phase:

(2) $$C_{t}=C_{0}+\frac{K_{u}}{\left(K_{e}\;+\;K_{growth}\right)}*C_{exp}*\left(e^{-\left(K_{e}\;+\;K_{growth}\right)*\left(t-t_{c}\right)\;}-e^{-\left(K_{e}\;+\;K_{growth}\right)*t}\right)\;$$

where *C_t_* is the concentration after *t* days of exposure (mg/kg dry body weight), *C*_0_ the background concentration (mg/kg dry body weight), *K_u_* the uptake rate constant (kg soil/kg organism/day), *K_e_* the elimination rate constant (day^−1^), *K_growth_* the growth rate constant (day^−1^), *C_exp_* the exposure concentration (mg/kg dry soil), *t* the exposure time (days), and *t_c_* the time (days) when the animals were transferred from contaminated to clean soil. 

Total and available (CaCl_2_-extractable) soil concentrations were used as the exposure concentration (*C_exp_*) to determine uptake and elimination rate constants.

The enchytraeids’ weight increased during the uptake phase, justifying the inclusion of a growth rate (*K_growth_*) in the kinetics equations to include any growth dilution effect. 

The bioaccumulation factor (BAF) (kg soil/kg organism), the ratio between organism’s concentration (mg/kg dry body weight) and soil concentration (mg/kg dry soil) at the equilibrium state, was estimated as the ratio between the uptake (*K_u_*) and the elimination rate (*K_e_*) constants:

(3) $$\mathrm{BAF}=\frac{K_{u}}{K_{e}}$$

Cr elimination half-life from the enchytraeids was calculated as:

(4) $${\mathrm{DT}}_{50}=\frac{\ln\left(2\right)}{K_{e}}$$

Models were fitted using internal concentrations in individual enchytraeids. All calculations were run in Excel for Microsoft 365 (Office 365 A1 Plus for faculty, V.2201) or SPSS 25.0. (IBM Corp. Released 2017. IBM SPSS Statistics for Windows, Version 25.0. Armonk, NY, USA: IBM Corp.) SigmaPlot 14.0 (2017, Systat software, Inc., Germany) was used to create the graphics presented. 

## 3. Results

### 3.1. Soil Analysis 

The total measured Cr concentration (mean ± SE) in the test soil was slightly lower than the nominal one, and was 145 ± 2.5 mg Cr/kg soil DW (80.6%), being constant throughout the exposure period (Table 1). The non-spiked soil contained 13.9 ± 0.6 mg Cr/kg soil DW. The CaCl_2_-extractable concentration in the spiked soil was 0.25 mg Cr/kg soil DW, which decreased by about 40% to 0.15 mg Cr/kg soil DW on day 1 and remained constant during the rest of the uptake phase (Table 1).

Both in the Cr-spiked and control soils, the soil pH slightly decreased during the 28-day test period, from 5.5 at the test start to 5.2 at the test end (Table 1).

### 3.2. Organism Weight 

**Figure 1:**
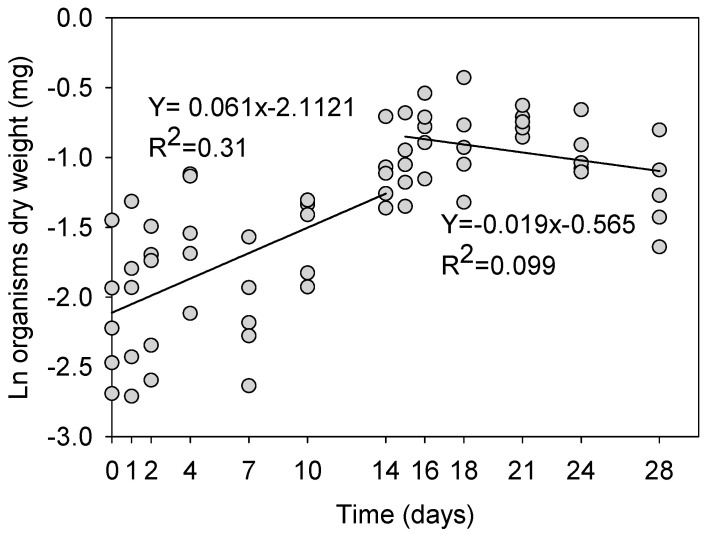
Dry body mass of *Enchytraeus crypticus* during test duration, including a 14-day uptake phase in LUFA 2.2 soil spiked at 180 mg Cr/kg dry soil followed by 14 days in clean LUFA 2.2 soil. Each dot represents an individual enchytraeid (*n* = 5 per sampling time). The lines show the fit of a linear regression model to the data of the uptake and elimination phases. Please note the logarithmic scale on the Y-axis.

Figure 1 shows the changes in dry body mass of the enchytraeids over the 28-day test period. The animals showed a steady increase in body mass during the uptake phase, with a *K_growth_* of 0.061 day^−1^ (R^2^ = 0.31), calculated using an exponential growth model. The enchytraeids showed a slight mass loss during the elimination phase, with a *K_growth_* of −0.019 day^−1^ (R^2^ = 0.099). These *K_growth_* values were used in the toxicokinetics calculations of Cr in the enchytraeids. 

### 3.3. Toxicokinetics

**Figure 2:**
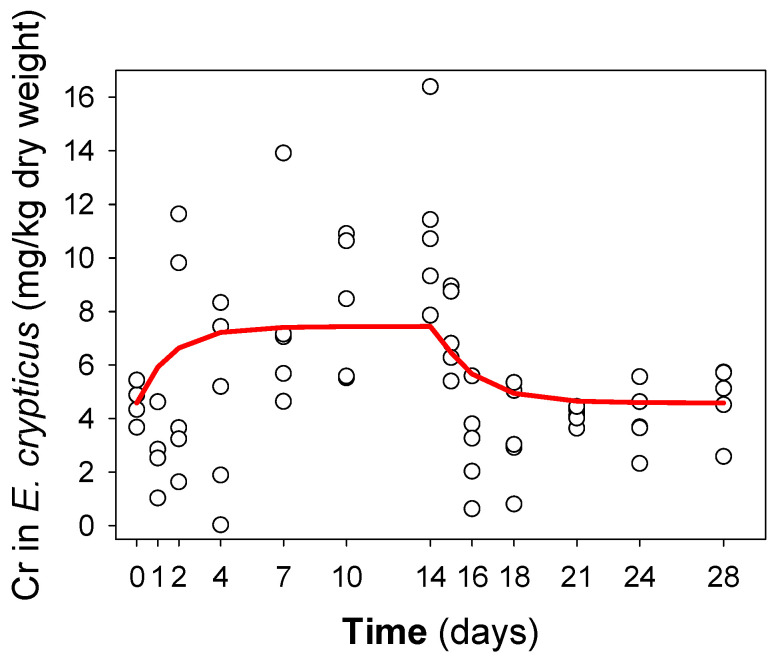
Uptake and elimination of Cr in *Enchytraeus crypticus* when exposed for 14 days in LUFA 2.2 soil spiked with 180 mg Cr/kg soil DW and then transferred to non-spiked soil, for a 14-day elimination period. The red line is the first-order one-compartment model fit (Equation (1)) based on total soil concentration (145 mg Cr/kg soil DW). The fit to the data included the animals’ growth rate, R^2^ = 0.24. Each dot is a measured replicate individual enchytraeid.

The average survival at the end of the exposure period was 88–94% for uptake and elimination phases, fulfilling the validity criteria (Table 1). An increase in Cr concentration with time was observed for *E. crypticus*, although showing higher variation in the internal concentration in the organisms at the uptake phase compared to the elimination (Figure 2). The mean coefficients of variation (CV ± SD) of the body Cr concentrations in the animals, calculated for each sampling day, were not significantly higher (*t*-test, *p* > 0.05) for the uptake phase (0.471 ± 0.231) than for the elimination phase (0.344 ± 0.199). 

At the start of the exposures, Cr concentration in *E. crypticus* was 4.6 ± 0.4 (mean ± SE) mg Cr/kg dry body weight. An increase in internal concentration was observed after exposure to 145 mg Cr/kg soil DW and an equilibrium in the internal concentrations was reached around day 10. The highest Cr concentration measured in a single enchytraeid was 16.4 mg Cr/kg dry body weight at day 14. 

Results of the parameter estimates and the model fit are summarized in Table 2. 

The uptake rate constant (*K_u_*) was 0.012 kg soil/kg organism/day and the elimination rate constant (*K_e_*) was 0.57 day^−1^. When organisms were transferred to clean soil, elimination occurred very quickly, with a DT_50_ of 1.2 days and the Cr concentration reaching the background level after 7 days. 

## 4. Discussion

A rapid accumulation of Cr was observed, with a steady state of the internal concentration in the organisms being reached after 10 days. After transferring the organisms to clean soil, the elimination was also fast, with body Cr concentrations returning to background levels within 7 days. Other studies also reported a fast accumulation and elimination of Cr in *Eisenia andrei* exposed to field-contaminated soils [27,39]. 

The present *E. crypticus* results showed a large variation in the measured internal Cr concentrations, and the kinetic parameters were not always significantly different from 0, mainly in the uptake phase. We applied more than one model to the data, choosing to present the one with the smaller confidence intervals and the best fit (R^2^). At the start of exposure, internal metal concentrations are dependent on both exposure concentration and time, but after equilibrium is reached, internal concentrations are only related to exposure concentration [29]. A large variation was also seen in other studies with metals and soil invertebrates [30,31,40], and can partly be related with the metal not being homogeneously distributed in the soil, but also with the differences in animal responses (uptake, metabolism, and excretion) [30,41]. Since the results of our study show low variation in soil concentrations, biological factors were probably more important. These factors may also include differences in individual animal behavior and variation in the age of the enchytraeids, which although being adult were taken from a non-synchronized culture. Despite the observed variation, the one-compartment model could be fitted to the data, making it possible to accurately describe the uptake and elimination of Cr in *E. crypticus* with time. We also tried another model which allowed for elimination of the initial body concentration (*C*_0_) in the enchytraeids (see Ardestani et al. [38]); however, this model did not provide a better fit to the data and in fact presented wider confidence intervals for the estimated *K*_u_ and *K*_e_ values.

For *E. crypticus*, the uptake and elimination rate constant were 0.012 kg soil/kg organism/day and 0.57 per day, respectively, with a BAF of 0.022 and DT_50_ of 1.2 days (when based on total soil concentrations). For *E. andrei* exposed to chromated copper arsenate (contaminated field soil and for 42 days, a longer period) [27], the *K_u_* values were 0.71 and 0.27 kg soil/kg organism/day for the highly and moderately contaminated sites, respectively, with elimination rate constants of 24.6 and 7.6 per day, respectively. These values were higher than the ones found for *E. crypticus*, but the BAFs were similar (0.029 and 0.036, respectively). Usually, the accumulation pattern of metals is both metal- and species-dependent [29]. The uptake rate constant is determined by the characteristics of the animal species, the toxic substance, and the environment (affecting metal bioavailability), while the elimination rate constant is mainly determined by the characteristics of the organism [29,42,43]. *Eisenia fetida* from two different locations, exposed (for 60 days) to sludge obtained from the drying beds of a wastewater treatment plant, showed BAF values of 0.61,0.68 for Cr [44]. These values are higher than those found for *E. crypticus* in this study but are also higher than the ones reported for earthworms in other studies. Soil properties like pH, CEC, OM, and clay content influence metal bioavailability, as do the metal, its concentration, the kind of pollution, and history [45,46]. Considering these factors and the fact that sludge was used could explain the higher BAF. Another study, exposing *E. andrei* for 3 weeks in artificial soil, reported internal concentrations between 0.8 and 18 mg Cr/kg dry weight for soil spiked with 10 and 1000 mg Cr/kg, respectively [28]. These internal Cr concentrations are in line with the highest internal concentration of 16.4 mg Cr/kg dry body weight found in this study, although at a lower exposure concentration. The BAF values for the lowest exposure levels (10,100 mg Cr/kg) ranged between 0.031 and 0.047 and were somewhat lower (0.016, 0.019) at the highest exposure levels (320 and 1000 mg Cr/kg). These BAFs are like those found in this study for enchytraeids exposed to 145 mg Cr/kg soil. In their study, Van Gestel et al. [28] concluded that earthworm concentrations exceeding 3 mg Cr/kg dry body weight were toxic, while 0.3 mg Cr/kg dry body weight was the control level. These results are different from ours because the background concentration of Cr in *E. crypticus* was much higher (4.6 mg Cr/kg dry body weight). The steady state and maximum concentrations of 7.6 and 16.4 mg Cr/kg dry body weight, however, already exceed the limit of toxicity reported by Van Gestel et al. [28] for *E. andrei*. The differences in results between studies can be partially explained by the different species and different exposure conditions used (field-contaminated and spiked soils, natural and artificial soils, different exposure scenarios), although the BAFs are a good source of comparison. 

The fast kinetics of Cr could be related to its essentiality to organisms and the way organisms can handle it. A similar pattern was observed for Cu, which is also an essential element, and less so for the non-essential elements Cd [30] and Ag [32]. As chromium is an essential element, it should be regulated by internal mechanisms that can differ, and the analysis of elimination patterns gives information on the possible detoxification mechanism in the organism [31]. For instance, when the shrimp *Palaemon elegans* was exposed to the essential element zinc, the elimination was increased to match the uptake, as the organisms are not able to control internalization through their body surface [47]. The crab *Carcinus maenas* actively restricts the internalization of zinc, and if the external concentration gets extremely high it will increase the internal concentration, but the excess zinc will be stored in a detoxified form in the hepatopancreas and in the exoskeleton [48]. 

The regulation of Cr in *E. crypticus* seems to be achieved by increasing the elimination rate to balance the uptake rate. Other studies report the same mechanism of elimination for soil organisms exposed to Cr [27,39].

The key turning point at which a chemical reaches a critical level, leading to adverse effects, can be understood through bioaccumulation studies. Since the exposure concentration used in this study (EC10 for reproduction effects) causes adverse phenotypic effects, it was expected that the internal concentration exceeded a critical level [24,49]. This is probably a key difference observed between *E. crypticus* and other Oligochaete species like *E. andrei*, which may explain differences observed in ECx and toxicity levels. The reason why *E. crypticus* background levels are significantly higher are not fully clear but should of course be considered in cross-species or taxa comparisons for Cr risk assessment. 

## 5. Conclusions

Chromium toxicokinetics in *E. crypticus* was well described by the one-compartment model. Chromium showed fast uptake and elimination kinetics in enchytraeids, reaching equilibrium after 10 days of exposure in contaminated soil and returning to background levels after around 7 days in clean soil. *E. crypticus* seemed able to regulate its internal body concentration by excreting Cr. Literature data on the toxicokinetics of Cr in soil organisms is limited and come mainly from tests with different exposure scenarios and species, but the BAF values were within similar ranges (at least in similar test designs). The evaluation of toxicity based on internal concentrations throughout time is a relevant way to assess the risk of metals, regardless of environmental factors, hence more toxicokinetics studies are recommended. 

## Figures and Tables

**Table 1:** Total and 0.01 M CaCl_2_-extractable Cr concentrations measured at days 0, 1, and 14 (mean ± SE) during the bioaccumulation test with *Enchytraeus crypticus* in LUFA 2.2 soil spiked with CrCl_3_. The table also includes the nominal concentration of Cr (mg Cr/kg soil DW), the soil pH (0.01 M CaCl_2_) measured at days 0 and 28, and the survival of the organisms at test end.

Nominal (mg Cr/kg Soil DW)	pH (CaCl_2_)		Measured Total (mg Cr/kg Soil DW) (Recovery)			CaCl_2_ Extractable (mg Cr/kg Soil DW)			Survival (%)
**Time (days)**	0	28	0	1	14	0	1	14	28
0	5.5	5.2	14 ± 1.2	-	13.8 ± 0.4	0.14 ± 0.0	-	0.12 ± 0.0	88 ± 5.8
180	5.5	5.2	147 ± 2.8 (81%)	139 ± 6 (77%)	148 ± 3.4 (82%)	0.25 ± 0.2	0.15 ± 0.0	0.16 ± 0.0	94 ± 5.8

(recovery), % recovery compared to nominal Cr concentration. -, not measured.

**Table 2:** Uptake and elimination rate constants of Cr estimated from the one-compartment model fit for *Enchytraeus crypticus* in LUFA 2.2 natural spiked soil. Values were calculated relating concentration in the enchytraeids to measured total and 0.01 M CaCl_2_-extractable concentrations in the soil. *K_u_*, uptake rate constant; *K_e_*, elimination rate constant; *C*_0_, background concentration in the animals; *C_exp_*, metal exposure concentration in spiked soil; *K_growth_*, growth rate of the animals during the 14-day uptake phase and during the 14-day elimination phase; BAF, bioaccumulation factor; DT_50_, time needed to eliminate 50%. [] brackets show the 95% confidence intervals.

Parameter	Total	CaCl_2_
***C_exp_*** (mg/kg dry soil)	145	0.15 *
***C*_0_** (mg/kg dry body wt.) (AV ± SE)	4.6 ± 0.4	
***K_u_*** (kg soil/kg animal/day)	0.012 [−0.06,0.3]	12 [−16.7,40.7]
***K_e_*** (day^−1^)	0.57 [−0.5,1.6]	0.57 [−0.5,1.6]
**DT_50_** (days)	1.2	
***K_growth_***_–uptake phase_ (day^−1^)	0.061	
***K_growth_***_–elimination phase_ (day^−1^)	−0.019	
**BAF**	0.022	21.1

* Average 0.01 M CaCl_2_ extractable concentration between day 1 and day 14 of the uptake phase.

## Data Availability

Data will be made available upon request to the corresponding author.
